# Reference values of physiological 18F-FET uptake: Implications for brain tumor discrimination

**DOI:** 10.1371/journal.pone.0230618

**Published:** 2020-04-17

**Authors:** Brigitte Fuenfgeld, Philipp Mächler, Dorothee R. Fischer, Giuseppe Esposito, Elisabeth Jane Rushing, Philipp A. Kaufmann, Paul Stolzmann, Martin W. Huellner

**Affiliations:** 1 Department of Nuclear Medicine, University Hospital Zurich, Zurich, Switzerland; 2 University of Zurich, Zurich, Switzerland; 3 Department of Radiology and Nuclear Medicine, Hospital St. Anna, Lucerne, Switzerland; 4 Department of Neurosurgery, University Hospital Zurich, Zurich, Switzerland; 5 Institute of Neuropathology, University Hospital Zurich, Zurich, Switzerland; Biomedical Research Foundation, UNITED STATES

## Abstract

**Purpose:**

The aim of this study was to derive reference values of 18F-fluoro-ethyl-L-tyrosine positron emission tomography (18F-FET-PET) uptake in normal brain and head structures to allow for differentiation from tumor tissue.

**Materials and methods:**

We examined the datasets of 70 patients (median age 53 years, range 15–79), whose dynamic 18F-FET-PET was acquired between January 2016 and October 2017. Maximum standardized uptake value (SUVmax), target-to-background standardized uptake value ratio (TBR), and time activity curve (TAC) of the 18F-FET-PET were assessed in tumor tissue and in eight normal anatomic structures and compared using the t-test and Mann-Whitney U-test. Correlation analyses were performed using Pearson or Spearman coefficients, and comparisons between several variables with Pearson’s chi-squared tests and Kruskal-Wallis tests as well as the Benjamini-Hochberg correction.

**Results:**

All analyzed structures showed an 18F-FET uptake higher than background (threshold: TBR > 1.5). The venous sinuses and cranial muscles exhibited a TBR of 2.03±0.46 (confidence interval (CI) 1.92–2.14), higher than the uptake of caudate nucleus, pineal gland, putamen, and thalamus (TBR 1.42±0.17, CI 1.38–1.47). SUVmax, TBR, and TAC showed no difference in the analyzed structures between subjects with high-grade gliomas and subjects with low-grade gliomas, except the SUV_max_ of the pineal gland (t-tests of the pineal gland: SUVmax: *p* = 0.022; TBR: *p* = 0.411). No significant differences were found for gender and age.

**Conclusion:**

Normal brain tissue demonstrates increased 18F-FET uptake compared to background tissue. Two distinct clusters have been identified, comprising venous structures and gray matter with a reference uptake of up to SUV_max_ of 2.99 and 2.33, respectively.

## Introduction

Positron emission tomography (PET) using the radiotracer 18F-fluoro-ethyl-L-tyrosine (18F-FET), an amino acid analogue, is becoming increasingly relevant in the initial assessment of primary brain tumors, and for differentiating tumor recurrences from post-therapeutic changes [1, 2]. In addition, 18F-FET-PET provides valuable input for treatment planning and therapy response assessment in patients, e.g. targeted tumor biopsy, surgery, and radiation therapy planning [3–5]. Several studies have documented the clinical feasibility and impact of 18F-FET, but its use is still limited to specialized centers [6–8].

Proton MR spectroscopy (MRS) has also been shown to be of value for grading and therapy response assessment of supratentorial gliomas [8–10]. Nevertheless, methodical challenges such as high susceptibility to artifacts in tumors close to air-filled spaces, variable acquisition techniques, volume averaging caused by voxel size and deviations in the calculation of metabolite ratios have limited widespread clinical implementation of MRS [8, 11].

Anatomical magnetic resonance imaging (MRI) currently serves as the imaging standard of reference for the non-invasive assessment and follow-up of brain tumors [12, 13]. However, combined 18F-FET-PET and MRI allows a more accurate delineation of tumor margins, along with a simultaneous identification of the region with the highest amino acid uptake, and hence a more accurate grading of brain tumors than MRI alone [14–17]. 18F-FET-PET was shown to aid the differentiation of low-grade gliomas (LGG) and high-grade gliomas (HGG). Several cut-off values of the target-to-background (TBR) ratio were established for this purpose [18, 19]. 18F-FET-PET uptake of inflammatory lesions appeared negligible in several studies, which is particularly useful when MRI results are equivocal [20–22].

However, normal anatomic brain structures such as deep grey matter, glandular structures, and venous vessels may exhibit 18F-FET uptake higher than normal parietal lobe, which serves as background reference tissue [6, 7, 15]. Currently, systematic data on 18F-FET characteristics of such normal cerebral structures and their relation to brain tumors is lacking in the literature. On the other hand, knowledge about normal morphological structures becomes even more important with the event of PET/MRI, which renders 18F-FET-PET imaging “more anatomical”. Without such knowledge, physiological uptake may be confounded with tumor-associated uptake. This can be the case in several brain tumors, particularly in those affecting the midline, such as corpora quadrigemina or basal ganglia tumors (i.e., papillary tumors of the pineal region, lymphomas, glioblastomas or oligodendrogliomas).

In our study, we aimed to derive reference values of 18F-FET uptake of normal brain and head structures in subjects in order to facilitate differentiation from tumor tissue.

## Materials and methods

All procedures performed were in accordance with the ethical standards of the institutional research committee and with the 1964 Helsinki declaration and its later amendments. Informed consent was obtained from all patients included in the study. This study was approved by the relevant ethical authorities (Kantonale Ethikkommission Zurich). Written consent was obtained with the approval number 2017–00152. Since September 2014, all patients are asked upon admission to give signed informed consent to allow the use of their health-related data for research. Patients who refused consent were not included in our study.

### Inclusion and exclusion criteria

For this retrospective study on subjects with brain tumors, data were collected from the picture archiving and communication system (PACS) and the clinical information system of our institution. All patients had received an 18F-FET-PET scan and an MRI scan between January 2016 and October 2017. The MRI exam was required to contain a minimum pulse sequence set T1-weighted (T1w) with and without contrast, T2-weighted (T2w), fluid-attenuated inversion recovery (FLAIR)-weighted image datasets. Exclusion criteria were incomplete 18F-FET-PET exam (e.g., incomplete dynamic acquisition or incomplete coverage of the brain), and any intervention between 18F-FET-PET and MRI scan.

### Image acquisition

All 18F-FET-PET were acquired using a Discovery VCT scanner (GE Healthcare, Waukesha, WI) or a Discovery 690 Standard scanner (GE Healthcare). According to the standardized protocol at our institution, all subjects were injected intravenously with 130 MBq of 18F-FET (mean 133 MBq, range 121–143 MBq) after a minimum of four hours of fasting to ensure standardized metabolic conditions. 18F-FET-PET data was acquired 20–40 minutes (min) after injection, using four 5 min frames. Emission data were corrected for randoms, dead time, scatter, and attenuation. Iterative reconstructions of the attenuation-corrected axial 18F-FET-PET datasets were done using a 128 × 128 pixels matrix (voxel spacing: 2.3438 * 2.3438 * 3.27).

Eight anatomic brain and head structures were analyzed, and the following 18F-FET-PET parameters were measured: standardized uptake value (SUV), target to background ratio (TBR), and time activity curve (TAC) pattern. SUVmax was defined as the average of the five hottest voxels of a particular structure. Five voxels were chosen since studies showed that averaging SUV from several voxel results in significantly lower variability between different measurements and therefore improves the accuracy and repeatability of SUV [23, 24].

TBR is the quotient of the SUVmax of a structure and the SUVmean of normal contralateral parietal lobe [25]. The mean 18F-FET uptake was measured using a crescent-shaped background VOI in the parietal lobe, including grey and white matter, as suggested by the joint EANM/EANO/RANO practice guidelines/SNMMI procedure standards [26].

Furthermore, the dynamic parameter TACs of 18F-FET uptake in the regions of interest were generated using a spherical VOI. For each VOI, the SUV_max_ of all four frames was measured. These values define three different slope patterns of the TAC: 1) wash-in, 2) plateau, and 3) wash-out. A plateau pattern was defined as a maximum change of +/-10%. A wash-in pattern refers to an increase by more than 10% during the aforementioned four 5 min frames, while a wash-out pattern refers to a decrease by more than 10% [19]. Symmetric brain structures were measured on the side with the higher 18F-FET uptake, to address potential confusion with tumor tissue. An exception was when tumor abutted this location. In this case, the contralateral structure was measured.

Data from ten patients was independently re-analyzed by a second reader, who used the same criteria, to test the inter-reader reliability of SUV, TBR, and TAC measurements. In addition, the first reader re-analyzed twenty datasets after a time interval of six weeks to assess intra-reader concordance. For all re-analyses, patients were selected using a random generator (https://www.ultimatesolver.com) in order to avoid selection bias.

Correlation with MRI was used to identify the aforementioned brain and head structures and to anatomically define the location of the brain tumor. MRI exams were conducted using different 3 Tesla MRI scanners at our institution.

### Image analysis

**Fig 1:**
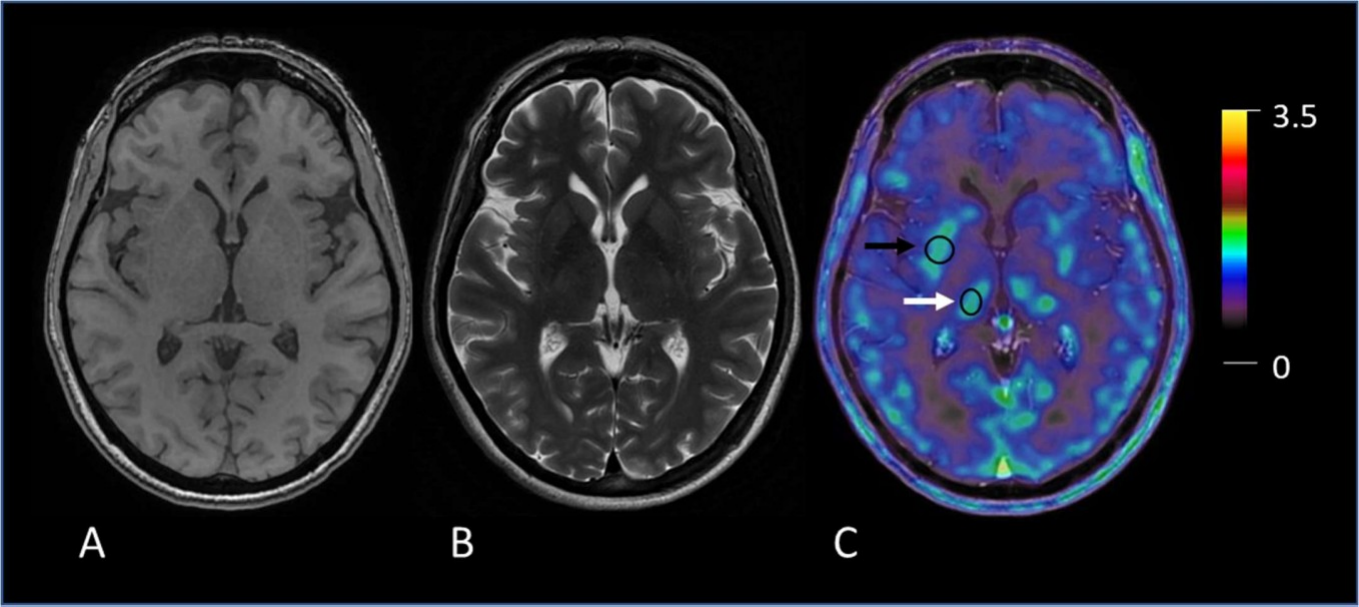
Example of measurement of 18F-FET uptake in anatomic brain structures. **A** T1-weighted (T1w) MR image, **B** T2-weighted (T2w) MR image, **C** fused image PET/MR image. Examples of two volumes-of-interest (VOI) for 18F-FET uptake calculation in physiological structures: putamen (black arrow) and thalamus (white arrow).

The MRI scan closest to the included 18F-FET-PET scan were analyzed for this study. Eight normal brain and head structures (caudate nucleus, cavernous sinus, pineal gland, putamen, sigmoid sinuses, superior sagittal sinus, temporal muscles, and thalamus) were investigated in each patient. Clinical pilot tests have shown that these structures have a high physiological 18F-FET uptake; examples are provided in Fig 1. In our study cohort, four tumors were located in the pineal gland region and three close to the superior sagittal sinus, hence no values were recorded from these structures in these subjects.

Analysis of 18F-FET-PET and MRI data were done using PMOD^®^ software (Version 3.8, PMOD technologies LLC, Zurich, Switzerland), which allows for separate, combined, and fused review of image datasets and a voxel-wise analysis.

### Statistical analysis

Descriptive analyses of patient characteristics and 18F-FET characteristics of tumors and anatomical structures were based on mean (with standard deviation), median (with range), and mode score (with percentage), depending on the particular scale of measurement. The distributions were inspected visually using histograms and boxplots; and if acceptable, the t-test was used for statistical comparison of ratio variables such as SUV_max_ and TBR. When the normal distribution was equivocal, the non-parametric U-test of Mann and Whitney was applied. A two-tailed *p*-value of less than 0.05 was defined as statistically significant. A possible connection between tumoral SUV_max_ and SUV_max_ of the eight analyzed structures was examined in two ways: First, the average tumoral SUV_max_ (2.9) was used as threshold in order to define two groups for the comparison of tumor and normal structures. In addition, six groups were binned by increasing tumoral SUV_max_.

Reference values were determined by a 95% reference interval using the mean (M) and two standard deviations (SD) of the 18F-FET uptake values (precisely, M ±1.96 SD). This calculation yields the 2.5th and 97.5th percentiles of the distribution, which serves as reference [27].

Statistical analyses were undertaken using the ‘Statistical Package for the Social Sciences’ (SPSS®, version 25, IBM, Armonk, NY). Differences among several variables were tested via crosstabulation with Pearson's chi-squared test (χ^2^). The Kruskal-Wallis test served as a non-parametric alternative test for the one-way analysis of variance (ANOVA). Statistical multiplicity, which is based on a set of simultaneous statistical inferences due to multiple comparisons (also known as the problem of multiple comparisons), was minimized with the Benjamini-Hochberg correction method [28].

## Results

Seventy brain tumor patients were included who were between 15 and 79 years-old (M±SD 49.8±16.2) and 42 were male (60.0%). 24 of 70 (34.3%) brain tumors were initial diagnoses, 46 (65.7%) were from follow-up examinations, e.g. suspected recurrence, of previously known and, partly, pretreated tumors. Primary brain tumors (65 of 70 patients, 92.8%) were classified according to the World Health Organization (WHO) 2016 system: 41 HGG (58.6%) and 24 LGG (34.3%) were verified histologically [29]. 12 of 70 patients (17.1%) patients had glioma suspected by MRI, but without histopathological confirmation before the 18F-FET PET scan. Subsequent clinical and radiologic follow-up with MRI and histopathology revealed LGG in all cases. Five of 70 patients (7.2%) had brain metastases. Demographic details, rationale for 18F-FET-PET scan, and tumor characteristics are given in Table 1.

**Table 1:** Patient demographics, tumors, and reason for 18F-FET-PET.

**Total patients (n)**					70
	Males (n (%))				42 (60%)
	Females (n (%))				28 (40%)
**Age [years] (mean (range))**					49.8 (15–79)
	10–19 years (n (%))				3 (4.3%)
** **	20–29 years (n (%))				6 (8.6%)
** **	30–39 years (n (%))				11 (15.7%)
** **	40–49 years (n (%))				12 (17.1%)
** **	50–59 years (n (%))				15 (21.4%)
** **	60–69 years (n (%))				16 (22.9%)
** **	70–79 years (n (%))				7 (10.0%)
**Histological diagnosis (n (%))**					
** **	**Gliomas according to WHO grading**				**65 (92.8%)**
	• WHO grade I				9 (12.9%)
		• low grade glioma (n = 9)			
	• WHO grade II				15 (21.4%)
		• low grade astrocytoma (n = 7)			
		• oligodendroglioma (n = 4)			
		• oligoastrocytoma (n = 2)			
		• pleomorphic xanthoastrocytoma (n = 1)			
		• ependymoma (n = 1)			
	• WHO grade III				19 (27.1%)
		• anaplastic astrocytoma (n = 12)			
		• anaplastic oligodendroglioma (n = 5)			
		• anaplastic oligoastrocytoma (n = 2)			
	• WHO grade IV				22 (31.4%)
		• primary glioblastoma (n = 20)			
		• secondary glioblastoma (n = 2)			
	**Metastases**				**5 (7.2%)**
**Indication for 18F-FET-PET scan (n (%))**					
	Initial diagnosis/grading of the tumor				16 (22.9%)
	Therapy planning/assessment of therapy response				18 (25.7%)
	Recurrence assessment				36 (51.4%)

18F-FET, 18F-fluoro-ethyl-L-tyrosine; WHO, World Health Organization

(here: WHO classification of tumors of the central nervous system (27)).

The median time interval between the 18F-FET-PET scan and the MRI scan was 29.5 days. Our analyses demonstrated low inter- and intra-reader reliability (with deviation of less than 10%).

The 18F-FET uptake (measured as SUV_max_) and the TBR of the eight examined structures and of the tumors are given in Table 2. SUV_max_ ranged from 0.72 in the caudate nucleus and 3.81 in the superior sagittal sinus, with a mean of 1.93 across all eight structures. The mean SUV_max_ of tumor tissue was 2.95 +/- 1.77 (median 2.67, range 0.85 to 9.40). Four tumors showed no 18F-FET uptake but were histologically classified as WHO grade II (n = 1), WHO grade III (n = 2) gliomas and metastasis (n = 1). The mean SUV_max_ of metastases (n = 5) was 2.57 ±1.7 (median 2.67, range 2.3 to 4.6). The mean TBR was 2.08 ±1.3 (median 2.14, range 0.6 to 3.3).

**Table 2:** 18F-FET-PET parameters in tumor tissue and normal brain and head structures.

18F-FET-PET	SUV_max_			TBR			TAC (n (%))			
parameter	Mean	Min	Max	Mean	Min	Max	Mean	1 wash-in	2 plateau	3 wash-out
Superior sagittal sinus	2.40	1.30	3.81	2.20	1.52	4.89	2.46	2 (2.9%)	32 (45.7%)	33 (47.1%)
Sigmoid sinuses	2.27	1.38	3.51	2.05	1.33	4.36	2.39	7 (10.0%)	29 (41.4%)	34 (48.6%)
Cavernous sinus	2.13	0.95	3.49	1.92	0.95	3.80	2.39	3 (4.3%)	37 (52.9%)	30 (42.9%)
Temporal muscles	2.18	1.32	3.52	1.97	1.03	3.45	2.11	5 (7.1%)	52 (74.3%)	13 (18.6%)
Pineal gland	1.64	0.93	3.24	1.47	0.73	2.31	1.85	21 (30.0%)	34 (48.6%)	11 (15.7%)
Putamen	1.61	0.74	3.01	1.41	1.07	1.85	1.67	28 (40.0%)	37 (52.9%)	5 (7.1%)
Caudate nucleus	1.59	0.72	3.39	1.40	0.97	1.86	1.56	33 (47.1%)	35 (50.0%)	2 (2.9%)
Thalamus	1.61	0.94	2.60	1.43	1.03	2.18	1.67	26 (37.1%)	41 (58.6%)	3 (4.3%)

N = 70; tumor tissue: n = 58, superior sagittal sinus: n = 67, pineal gland: n = 66 (If tumors were abutting the pineal gland or the superior sagittal sinus, no values were recorded from these structures.)

18F-FET, 18F-fluoro-ethyl-L-tyrosine; Max, maximum; Min, minimum; SUV_max_, maximum standardized uptake value; TAC, time activity curve; TBR tumor-to-brain or target-to-background standardized uptake value ratio.

Reference values for the eight brain and head structures, derived as described above, ranged from 1.15 to 2.03 (caudate nucleus) and 1.99 to 2.81 (superior sagittal sinus). All values are given in Table 3.

**Table 3:** 18F-FET-PET reference values for normal brain and head structures.

18F-FET-PET	SUV_max_		reference values (M±2 SD)	
parameter	Mean	SD	lower limit	upper limit
Superior sagittal sinus	2.40	0.41	1.99	2.81
Sigmoid sinuses	2.27	0.48	1.79	2.75
Cavernous sinus	2.13	0.49	1.64	2.62
Temporal muscles	2.18	0.42	1.76	2.60
Pineal gland	1.64	0.47	1.17	2.11
Putamen	1.61	0.43	1.18	2.04
Caudate nucleus	1.59	0.44	1.15	2.03
Thalamus	1.61	0.36	1.25	1.97

N = 70; tumor tissue: n = 58, superior sagittal sinus: n = 67, pineal gland: n = 66 (If tumors were abutting the pineal gland or the superior sagittal sinus, no values were recorded from these structures.)

18F-FET, 18F-fluoro-ethyl-L-tyrosine; M, mean; SD, standard deviation; SUV_max_, maximum standardized uptake value.

HGG had a mean of SUV_max_ in the tumor tissue of 3.53 ±1.8 (median 3.77, range 1.5 to 9.4), with a maximum of 9.4 found in a case of glioblastoma in a 61-year-old man. The mean TBR of HGG tumor tissue was 2.98 ±1.1 (median 2.99, range 1.2 to 5.9), while TBR of two lesions was below 1.5. LGG exhibited a mean SUV_max_ of 1.97±1.5 (median 2.25, range 0.85 to 4.6) with a maximum of 4.6 in a low grade glioma from a 51-year-old woman. The mean TBR of tumor tissue in LGG was 2.23 ±0.9 (median 2.03, range 0.92 to 4.0). Notably, SUV_max_ and TBR of the tumor tissue differed significantly between HGG and LGG (independent samples t-test (SUV_max_: t(60) = -3.613, p = .001; TBR: t(54) = -2.512, p = .01). SUV_max_ of the pineal gland was significantly lower in LGG subjects than in HGG subjects (independent samples t-test, t(59) = -2.357, p = 0.022). However, no significant differences were found between HGG and LGG subjects in the seven remaining brain structures. Details are given in Table 4.

**Table 4:** 18F-FET-PET parameters in high-grade and low-grade gliomas.

	18F-FET-PET parameter	HGG (n = 41)	LGG (n = 24)	p-value
Tumor tissue	SUV_max_ (mean ± SD)	3.53±1.8	1.97±1.5	0.001**
	TBR_max_ (mean ± SD)	2.98±1.1	2.23±0.9	0.015*
	TAC (mean rank)	11.00	9.20	0.441
Superior sagittal sinus	SUV_max_ (mean ± SD)	2.42±0.4	2.41±0.41	0.881
	TBR_max_ (mean ± SD)	2.12±0.4	2.39±0.7	0.077
	TAC (mean rank)	32.36	30.15	0.589
Sigmoid sinuses	SUV_max_ (mean ± SD)	2.32±0.5	2.24±0.5	0.548
	TBR_max_ (mean ± SD)	1.99±0.4	2.21±0.7	0.104
	TAC (mean rank)	34.50	30.44	0.356
Cavernous sinus	SUV_max_ (mean ± SD)	2.13±0.6	2.12±0.5	0.948
	TBR_max_ (mean ± SD)	1.83±0.5	2.09±0.6	0.056
	TAC (mean rank)	31.98	34.75	0.518
Temporal muscles	SUV_max_ (mean ± SD)	2.25±0.4	2.13±0.4	0.255
	TBR_max_ (mean ± SD)	1.93±4.2	2.09±0.5	0.210
	TAC (mean rank)	32.12	34.50	0.532
Pineal gland	SUV_max_ (mean ± SD)	1.77±0.5	1.48±0.3	0.022*
	TBR_max_ (mean ± SD)	1.52±0.4	1.43±0.4	0.411
	TAC (mean rank)	31.08	30.86	0.961
Putamen	SUV_max_ (mean ± SD)	1.66±0.4	1.53±0.5	0.292
	TBR_max_ (mean ± SD)	1.40±0.2	1.52±0.2	0.690
	TAC (mean rank)	33.48	32.19	0.766
Caudate nucleus	SUV_max_ (mean ± SD)	1.63±0.4	1.53±0.5	0.409
	TBR_max_ (mean ± SD)	1.38±0.3	1.42±0.3	0.459
	TAC (mean rank)	32.55	33.77	0.775
Thalamus	SUV_max_ (mean ± SD)	1.63±0.3	1.55±0.4	0.383
	TBR_max_ (mean ± SD)	1.39±0.2	1.47±0.2	0.158
	TAC (mean rank)	35.90	28.04	0.63

N = 70; tumor tissue: n = 58, superior sagittal sinus: n = 67, pineal gland: n = 66 (If tumors were abutting the superior sagittal sinus or the pineal gland, no values were recorded from these structures.). SUV_max_ and TBR: independent samples t-test, TAC: Mann-Whitney U-test

Sig. (two-tailed): *p < .05, **p < .01.

18F-FET, 18F-fluoro-ethyl-L-tyrosine; HGG, high-grade glioma; LGG, low-grade glioma; SD, standard deviation; SUV_max_, maximum standardized uptake value; TBR_max_, maximum tumor-to-brain or target-to-background standardized uptake value ratio.

In addition, it was found that the higher the tumoral SUV_max_, the higher the SUV_max_ of the eight normal structures in our study. Mean SUV_max_ and TBR values showed no significant differences according to age (univariate analysis over 7 age decades (Table 1) as well as non-parametric rank Kruskal-Wallis test) or gender (independent samples t-test).

**Fig 2:**
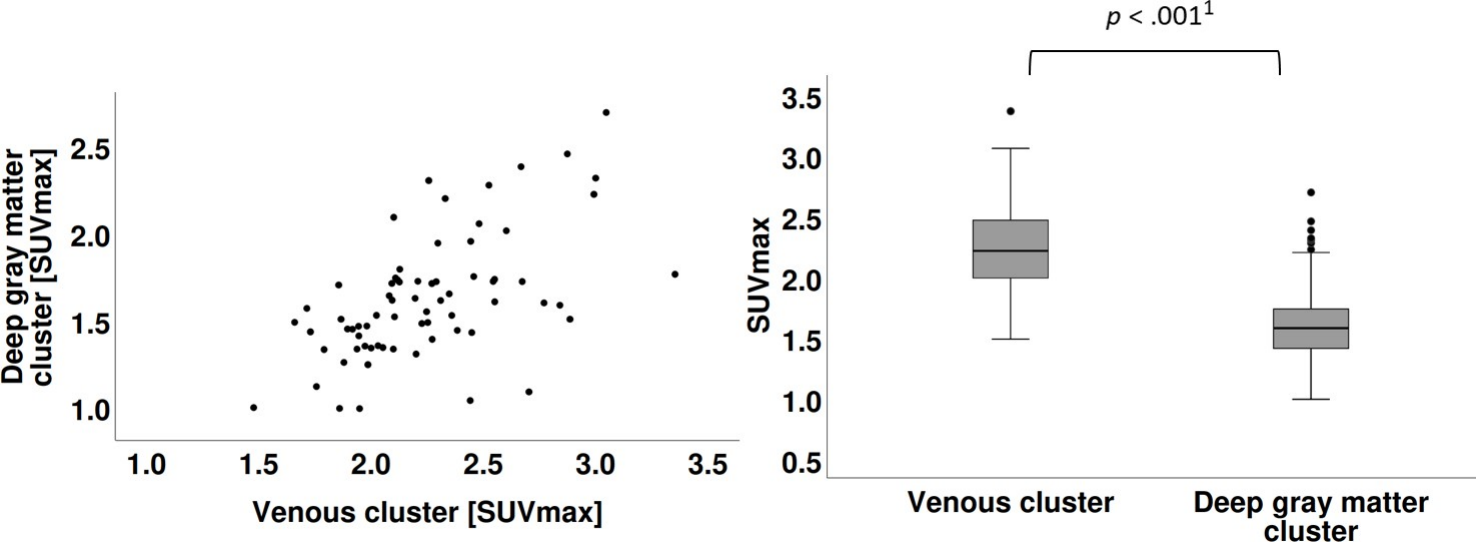
Scatterplots of SUV_max_. **A** Scatterplot of SUV_max_ of venous cluster with SUV_max_ of deep gray matter cluster. **B** Significant difference in the SUV_max_ were found for venous cluster (M = 2.25, SD = 0.37) and deep gray matter cluster (M = 1.61, SD = 0.36), paired sample t-test, ^1^*p* = 1.0129E-24.

**Fig 3:**
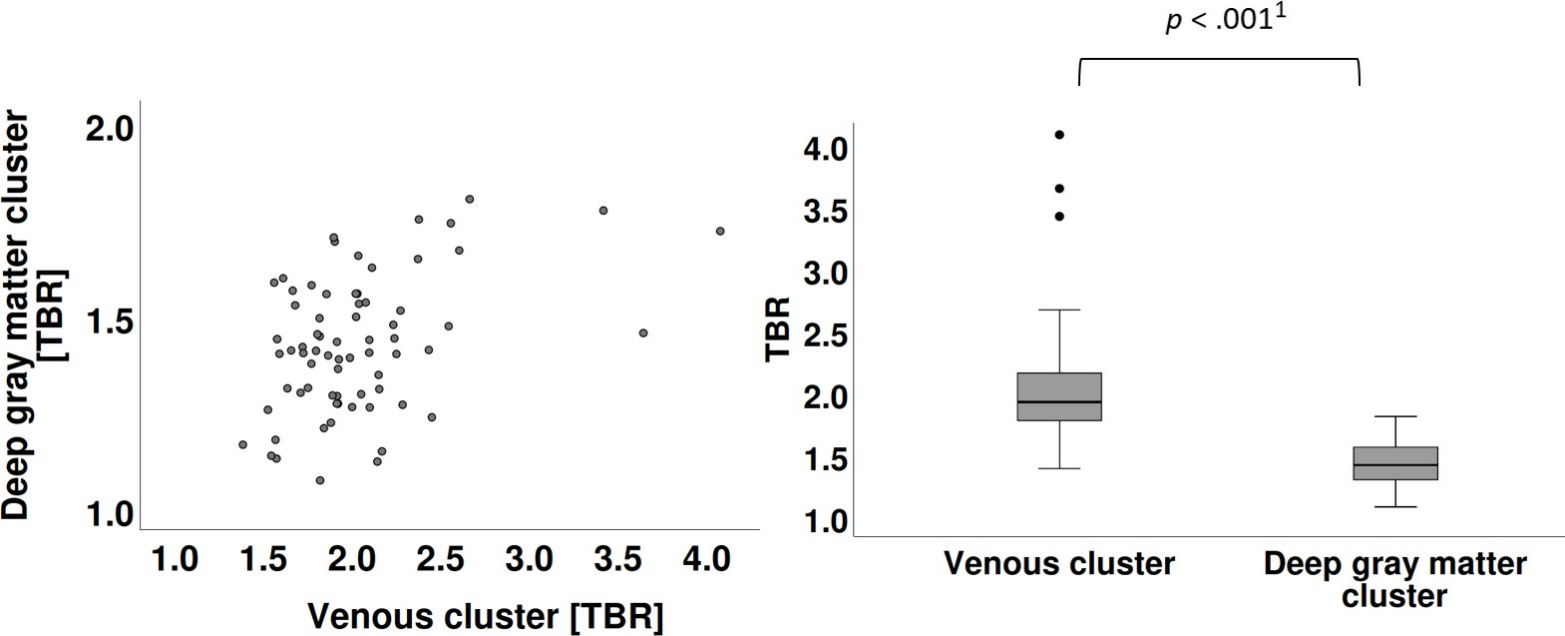
Scatterplots of TBR. **A** Scatterplot of TBR of venous cluster with TBR of deep gray matter cluster. **B** Significant difference in the TBR values were identified for venous cluster (M = 2.03, SD = 0.46) and deep gray matter cluster (M = 1.42, SD = 0.17), paired sample t-test, ^1^ *p* = 6.312E-19.

Comparison of SUV_max_ (Fig 2) and the TBR (Fig 3) of the eight investigated structures revealed two clusters, each consisting of four structures: The first cluster comprised the three venous sinuses and the temporal muscles (hereafter referred to as the “venous cluster”) had a group mean of 2.25 (95% confidence interval [CI], 2.16–2.33; SD = 0.37). The second cluster consisted of the pineal gland, putamen, caudate nucleus, and thalamus (hereafter referred to as the “deep gray matter cluster”) had a group mean of 1.61 (95% CI, 1.52–1.70; SD = 0.36). The differences in SUV_max_ of the two clusters were significant following a paired sample t-test (t(69) = 15.869, *p* < .001).

**Fig 4:**
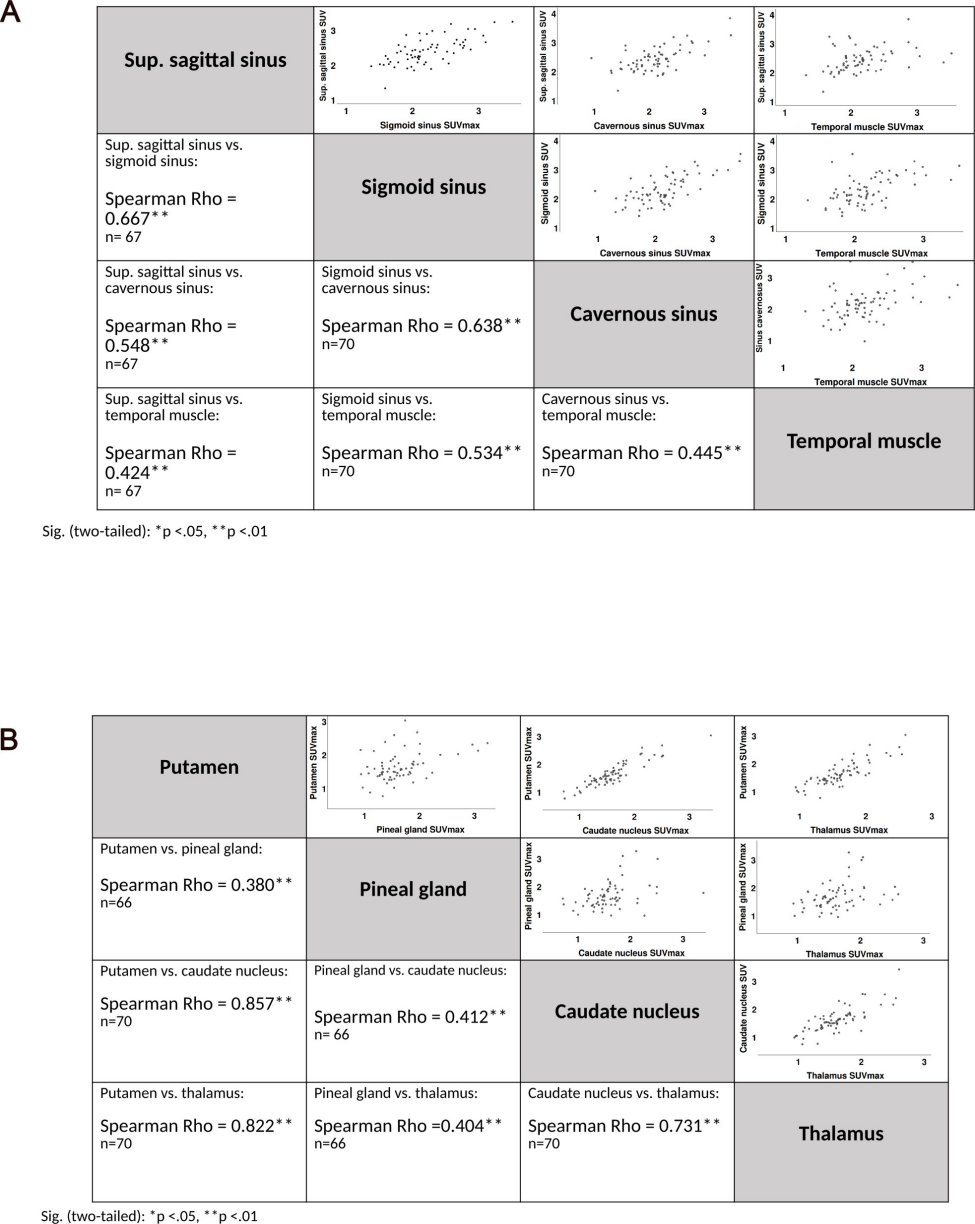
Intra-cluster correlations. **A** Intra-cluster correlations of the venous cluster vary between 0.42 and 0.67, all highly significant. **B** Intra-cluster correlations of the deep gray matter cluster vary between 0.38 and 0.82, all highly significant. Sig. (two-tailed): **p < .01. Spearman Rho rank correlation applicable for non-parametric distribution in the scatter plot. Strength of correlation < 0.3 = no correlation, 0.3–0.5 weak, 0.5–0.8 moderate, > 0.8 strong correlation [30].

The venous cluster showed a group TBR mean of 2.03 (95% CI, 1.92–2.14; SD = 0.46) and deep gray matter cluster, a group mean of 1.42 (95% CI, 1.38–1.47; SD = 0.17). Differences in the TBR values for the venous cluster and deep gray matter cluster were significant (paired sample t-test: t(69) = 12.221, *p* < .001). The CIs for both—SUV_max_ and TBR mean of the clusters—were mutually exclusive and narrow with less than 5% deviation from the mean of venous cluster and slightly above a 5% from the mean of deep gray matter cluster. Intra-cluster correlations (Pearson *r* or Spearman Rho with a non-parametric distribution in the scatter plot) were all highly significant with *p* < .01. Details are given in Fig 4.

**Fig 5:**
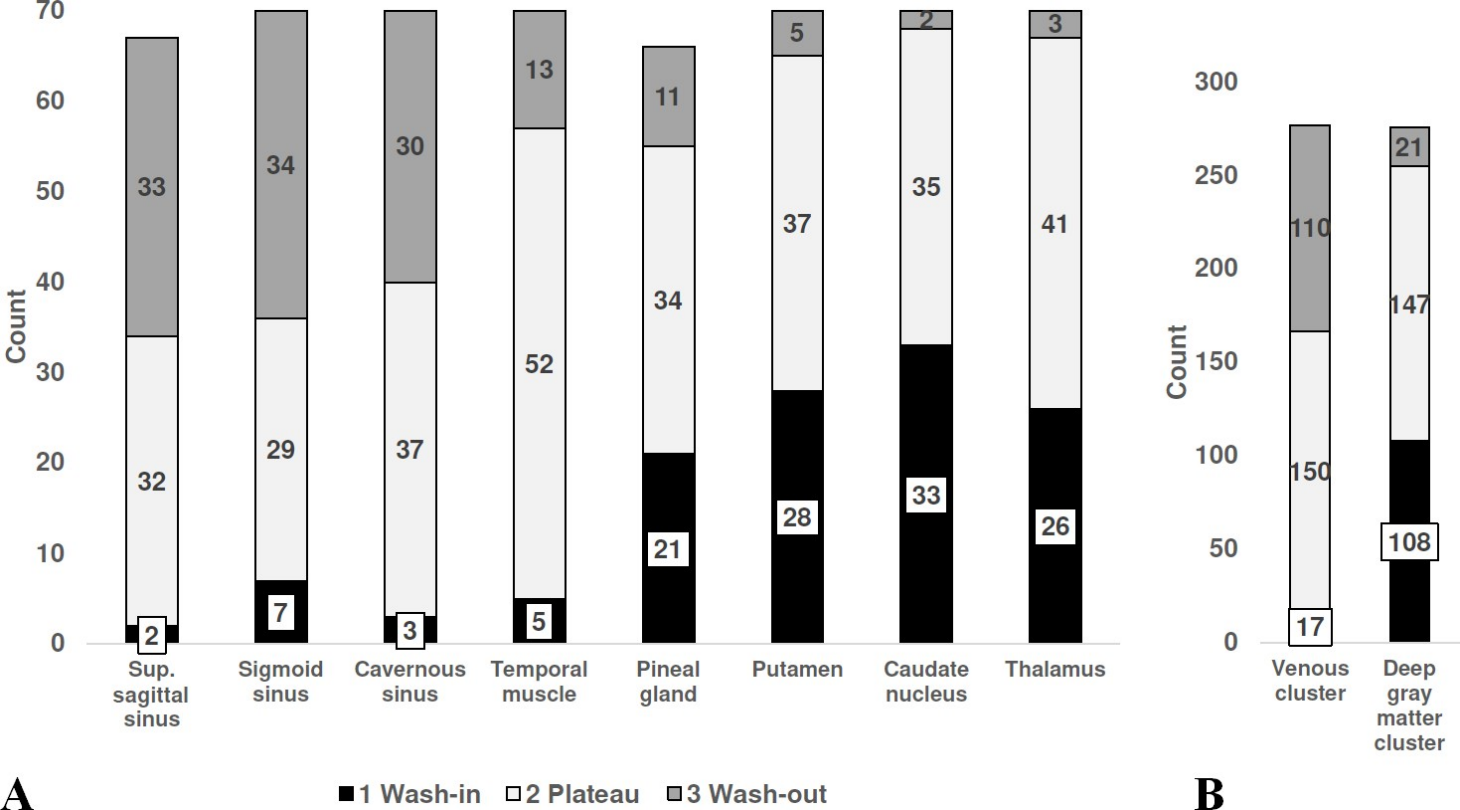
Time activity curves. **A** The three different patterns of the TAC of 18F-FET uptake (1 wash-in, 2 plateau, and 3 wash-out) were unequally distributed among the eight structures. **B** Distribution of the TAC pattern reflects the two clusters venous cluster and deep gray matter cluster. The crosstabulation Pearson’s chi-squared test suggests that the two clusters are not related regarding TAC (χ2 (2) = 126.74, p < .001). N = 70; superior sagittal sinus: n = 67, pineal gland: n = 66 (If tumors were in the superior sagittal sinus or in the pineal gland, no values were recorded from these structures).

All eight structures exhibited three predefined TAC patterns (wash-in, plateau, and wash-out), however, with differing distribution of the patterns: The share of wash-in varied from 3% (superior sagittal sinus) to 47.1% (caudate nucleus), the plateau from 41.4% (sigmoid sinuses) to 74.3% (temporal muscles), whereas the proportion of wash-out reached from 2.9% (caudate nucleus) to 49.3% (superior sagittal sinus). There was no significant difference in the distribution of TAC between patients with HGG versus LGG (Mann-Whitney U-test, details in Table 3). In addition, the distribution of the TAC patterns reflected the two clusters. Crosstabulation Pearson’s chi-squared test showed that the clusters differ significantly regarding TAC (χ^2^(2) = 126.74, *p* < .001). Details are given in Fig 5.

## Discussion

18F-radiolabeled amino acid FET has become a valuable and clinically established tracer for PET imaging in patients with brain tumors [2, 31, 32]. Sensitivity of 18F-FET-PET for neoplastic brain tissue is high, while specificity is low, because 18F-FET also accumulates in non-tumoral tissue [33–37]. In our study, we systematically characterized 18F-FET-PET features in eight anatomic brain and head structures (caudate nucleus, cavernous sinus, pineal gland, putamen, sigmoid sinuses, superior sagittal sinus, temporal muscles, and thalamus) that showed prominent accumulation in clinical pilot tests in tumor patients, owing to their comparably high physiological 18F-FET uptake.

Literature on 18F-FET-PET uptake of non-neoplastic lesions is limited and mainly describes incidental findings such as aneurysms and abscesses, while some retrospective data exists on demyelinating lesions [21, 38–40]. Little is known about 18F-FET imaging characteristics of normal brain structures. Some studies have investigated normal brain tissue, reporting rather homogenous results within white matter and superficial gray matter. Separate brain regions or distinct structures were not analyzed [41, 42].

We found that all eight normal structures exhibited SUV_max_ and TBR values considerably above background. For TBR in our study, a threshold value of 1.5 was considered indicative of increased 18F-FET uptake. This threshold is based on several former studies, in particular, a biopsy-controlled study from Pauleit et al. (2005), where a TBR above 1.5 discriminated neoplastic from normal brain tissue [38, 42, 43]. Our study population exhibited values both over and under the TBR threshold values of 1.5 in seven structures and only the superior sagittal sinus exhibited a TBR consistently above this threshold.

On average, 18F-FET uptake in anatomic structures was lower than in tumor tissue. However, the range of 18F-FET uptake from tumor and non-tumor overlapped to a great extent, precluding reliable discrimination based on TBR alone [44]. These findings indicate that 18F-FET-PET sensitivity for tumor tissue is high, but not strongly specific. In addition, blood brain barrier breakdown may not be required for exhibiting ‘pathological’ 18F-FET uptake [7, 33, 45, 46]. Admittedly, the highest TBR in our study was found in the superior sagittal sinus, which is outside the blood brain barrier. Future reference validation might take into account such vascular brain structures that provide information on the blood pool, in addition to the established parietal lobe brain tissue.

In addition, our study provides some evidence that 18F-FET uptake in normal structures is actually linked to tumoral 18F-FET uptake. The higher the tumoral SUV_max_, the higher was the SUV_max_ of the eight normal structures. Results of both analyses on this issue were consistent, with the course of SUV_max_ in the subgroups comparable to our entire sample, thus confirming the robustness of our data. However, the increase from group to group is small and is based on a limited number of patients per group. In addition, the size of the groups is unequally distributed, especially the lowest and highest groups are small, so that the outliers affect the mean. Therefore, this result warrants further substantiation by studies with a larger number of patients, including stratification according to different types of primary and secondary tumors. Additional research might illuminate if there is a possible interrelation influenced by tumoral SUV_max_, either related to overall higher SUV_max_ in a particular patient's brain or another factor. Nevertheless, the finding that the uptake of certain normal structures was high, with higher uptake in brain tumors substantiates the assumption that blood pool may play an important role in the degree of tumoral uptake. Tumoral 18F-FET uptake may be consistently overestimated if only combined parietal lobe grey and white matter is considered as the reference [26]. Future studies are encouraged to investigate the use of other referential tissues such as basal ganglia.

We have found no significant difference of 18F-FET-PET parameters between HGG and LGG cases except for the pineal gland and the intrinsic tumors values. These findings are in line with the large overlap of HGG and LGG regarding SUV_max_ and TBR in different studies [42, 44]. No significant differences were found for gender and age. Patients are not equally distributed among age groups, e.g., a few patients were in age range of 10–30 years and above 70 years. Therefore, conclusions about age groups need to be drawn with caution.

Another major result of our study is that the eight analyzed structures constitute two clusters by SUV_max_, TBR, and TAC. The composition of the two clusters is similar in all of the above parameters: The first cluster (i.e., venous cluster) contains the three sinuses and temporal muscles, which are extraaxial structures not subject to the blood brain barrier. The second cluster (i.e., deep gray matter cluster) is composed of the caudate nucleus, pineal gland, putamen, and thalamus, which are intraaxial structures isolated by the blood brain barrier, except for the pineal gland. There was a marked difference between the mean and range of the two clusters. The CI of the means were mutually exclusive and with narrow ranges, both being indicators for a relevant statistical approximation. The two clusters suggest reference values for SUV_max_ (venous cluster: 2.25±0.08, deep gray matter cluster: 1.61±0.09) and for TBR (2.03±0.11 and 1.42±0.05, respectively) (Figs 2 and 3).

The composition of the clusters indicates that the difference in 18F-FET uptake is influenced by blood flow. The sinuses and the muscles show a comparably high 18F-FET uptake, in line with the common finding of positive correlation between cerebral blood volume and amino 18F-FET uptake with 11C-methyl-L-methionine (11C-MET) [47]. The amino acid 18F-FET is not metabolized or incorporated into proteins, but its accumulation in tissue is due to the transport mediated by the L-type amino acid transport system [6, 48, 49]. Moreover, the distribution of TAC patterns among the two clusters, with a higher portion of wash-out in the cluster of the sinuses and muscles, is in line with findings in the literature, although only a part of the variance of 18F-FET wash-out may be explained by perfusion [50, 51].

The low specificity of 18F-FET-PET can present challenges such as inability to discriminate between tumoral and normal tissue in specific brain structures. 18F-FET uptake values of the eight brain and head structures acquired by the M ± 2 SD approach may serve as reference values in a clinical setting. Two clearly distinct clusters were identified and comprise venous structure and gray matter. The reference uptake of the two clusters reaches 2.99 and 2.33, respectively.

In clinical practice, it is recommended that uptake values and imaging characteristics reported for gliomas should take into account the background activity of individual adjacent tissue for standardization [52]. Further studies may repeat our analysis with PET/MRI, which is increasingly available. PET/MRI allows for multiparametric imaging at the same time by combining conventional MRI, advanced MRI, and PET imaging, all images acquired under the same physiological conditions and the same iso-center for optimal realignment. However, the issue of attenuation correction is not fully solved [53–55].

### Limitations

We acknowledge that our study involved only patients with brain tumors; there was no normal control group. It might have been beneficial to study healthy subjects in order to avoid potential confounding factors. However, we refrained from this option because of ethical considerations. Additionally, relevant structures in our analysis were thoroughly verified on MRI to confirm the absence of tumor tissue there and in their vicinity. Another limitation of our study is that most patients had undergone surgery and/or biopsy (some repeatedly), together with different types of radiotherapy and chemotherapy, which may have altered 18F-FET uptake.

## Conclusion

This study aimed to examine the uptake characteristics of 18F-FET measured by SUV_max_, TBR, and TAC within normal structures of the brain and head in tumor patients. We found that the investigated eight structures exhibit a systematic 18F-FET uptake within the range of tumor tissue and form two distinct clusters: the cluster with well-perfused structures sinuses and muscles show higher 18F-FET uptake than the cluster comprising intraaxial structures. Reference values for 18F-FET uptake of the two clusters reach SUV_max_ of 2.99 and 2.33, respectively.

To our knowledge, this is the first study to systematically evaluate normal brain and head structures. Our findings confirm that the specificity of 18F-FET-PET is limited by the inability to differentiate tumor tissue from normal structures, based only on their uptake characteristics [7, 33]. Future work is indicated to examine the additional use of blood pool for reference purposes in order to standardize 18F-FET uptake.

## Supporting information

S1 Data (XLSX)
